# Targeting apoptotic anticancer response with natural glucosinolates from cell suspension culture of *Lepidium sativum*

**DOI:** 10.1186/s43141-023-00511-y

**Published:** 2023-05-02

**Authors:** Mona M. Ibrahim, Marwa M. Mounier, Shawky A. Bekheet

**Affiliations:** 1 Department of Plant Biotechnology, Biotechnology Research Institute, National Research Centre, Cairo, 12622 Egypt; 2 Department of Pharmacognosy, Pharmaceutical and Drug Industries Research Institute, National Research Centre, Cairo, 12622 Egypt

**Keywords:** *Lepidium sativum*, Cell culture, Precursors, Cell line, Apoptotic protein

## Abstract

**Background:**

Finding natural products with anticancer activity is an effective strategy to fight this disease. In this respect, *Lepidium sativum* or garden cress (family *Brassicaceae*) has been widely used worldwide for its wide therapeutic application, including anticancer and chemoprotective agents. Plant tissue culture techniques hold great promise for natural product enhancement without any climatic boundaries. In this study, glucosinolates and petroleum ether fractions were isolated from in vitro cell cultures and used against different carcinoma cell lines to investigate their anticancer potential.

**Methods:**

In this study, callus cultures from leaf and root explants were initiated, cell suspension cultures were established, and cell growth and viability profiles were characterized. Different amino acids were added as precursors to the cell suspension cultures to enhance glucosinolates accumulation. Gas chromatography–mass spectrometric analysis (GC–MS) of glucosinolates and petroleum ether fractions was performed, and all fractions were tested against different carcinoma cell lines.

**Results:**

The findings clarified that the maximum callus initiation percentage was obtained in the medium containing 1.0 mg/l 2,4-dichlorophenoxy acetic acid (2,4-D) + 1.0 mg/l kinetin (Kin) (C1). The viable cell number of cell suspension cultures from leaves and roots increased until it reached the maximum values on day 15. Adding tyrosine and methionine to the cell suspension cultures was the most influential and recorded high glucosinolate percentages. 1H-Cyclopenta (b) pyridine-3-carbonitrile-4,5,6,7-tetrahydro-2-methylthio-4-spirocyclohexane was the main glucosinolate compound found in tyrosine-treated leaf suspension (GLT). Fifteen compounds were detected in the petroleum ether fraction in both cell suspensions initiated from the leaf and root (OL and OR). The major compounds were benzene-1,3,5-trimethyl (12.99%) in root cell suspension (OR), and benzene-2-ethyl-1,4-dimethyl (10.66%) in leaf cell suspension (OL). All glucosinolate extracts demonstrated significant anticancer activity against the prostate (PC3), lung (A-549), colorectal (caco2), and liver (HepG2) cell lines. Glucosinolates extracted from leaf cell suspension (GL) were the most active on the hepatocellular carcinoma cell line (HepG2) among all remaining glucosinolate extracts. Treated hepatocellular carcinoma with an IC_50_ of GL extract (47.5 ug/ml) upregulates pro-apoptotic BAX and downregulates anti-apoptotic BCL2, which disrupts the BAX/BCL2 ratio, leading to activation of caspase 3 inside treated HepG2 cells.

**Conclusions:**

The anticancer action of the GL extract was validated by the cell cycle study of its glucosinolates, which successfully promoted apoptosis and reduced hepatocellular growth by causing S-phase arrest.

## Background

*Lepidium sativum*, or garden cress (family *Brassicaceae*), is an edible herb cultivated in various parts of the world. It is considered a standing herbaceous annual plant that grows from 15 to 45 cm in height and has its own small white flowers along with broad or obovate pods emarginated at the apex and winged [1]. It has been used to treat various clinical problems [2] and is a highly nutritious plant [3]. This is due to the high concentration of functional ingredients in this plant, such as tocopherol, phenolic compounds, nitrogen compounds, terpenoids, and vitamin E, all of which have strong antioxidant activity [4]. Furthermore, it contains sulfur-containing compounds, particularly glucosinolate compounds (GLSs), which have various biological effects, including potent anti-proliferative activity against some carcinoma cell lines via ROS in mitochondrial-mediated apoptosis [5, 6].

Garden cress has been declared to have various clinical values in traditional medicine, including for inflammatory diseases such as diabetes mellitus, arthritis, and hepatitis [7, 8]. Cancer is a substantial public health threat in developed and developing countries, leading to high mortality rates worldwide. Various chemotherapy drugs have been used for cancer reduction, but it is advised to restrict their usage because of the harmful side effects [9]. Plant extracts, as a natural therapy, are helpful in fighting cancer. It has been used as a source of medicine since ancient times and in various contexts.

Plant biotechnology techniques fulfill the output of plant material and natural products to some extent if all factors that achieve this goal are controlled. Plant cell culture introduces many optimization strategies, such as precursor feeding, media, and cultivation modifications [10]. Several attempts have been made to produce GLSs in cell cultures, such as GLSs production in *Arabidopsis thaliana* cell cultures [11].

In the previous study, glucosinolate compounds were detected in callus cultures, and these extracts were effective against three human carcinoma cell lines [12]. The current research was designed to establish and produce cell suspension cultures from leaf and root explants. Different amino acids (precursors) addition strategy have increased GLSs content in leaf and root suspension cultures. Also, components in the oil of the petroleum ether fraction were identified for both suspension cultures (leaf and root). Glucosinolate extracts and oil fractions from cell suspension cultures were tested against various carcinoma cell lines, followed by cell cycle analysis and the identification of various apoptotic parameters.

## Methods

### Source of reagents and chemicals

MS-medium, gelrite, 2,4-dichlorophenoxy acetic acid (2,4-D), kinetin, benzyladenine, trypan blue, L-phenylalanine, L-tyrosine, L- methionine, and L-cysteine purchased from Biotech Serve Co. Potassium penicillin, RPMI 1640 medium, streptomycin sulfate, amphotericin B, L-glutamine, doxorubicin, MTT salt, sodium dodecyl sulfate (SDS), biotin-conjugated antibody, streptavidin-HRP, streptavidin conjugated to horseradish peroxidase, and propidium iodide (PI) were brought from Sigma, Aldrich. DMSO, petroleum ether, ethanol, Tween 20, and dichloromethane were purchased from Omega for Trading of Solvents.

### Plant material and explant preparation

Seeds of* L. sativum* were immersed in 70% ethanol for 2–3 min, then rinsed three times in sterile distilled water. The seeds were then sterilized for 20 min in 20% commercial Clorox (5% NaOCl) containing 0.5% Tween 20. After rinsing three times with sterile distilled water, seeds were cultured on MS medium (Murashige and Skoog, 1962) containing 3% (w/v) sucrose and solidified with 0.2% (w/v) gelrite. The culture medium was adjusted to pH 5.8. The cultures were incubated in a growth room at 26 ± 2 °C and kept under a 16-h photoperiod of fluorescent, 45 μmol cool white light tubes, and an 8-h dark.

### In vitro callus culture initiation

Explants of leaf and root (approximately 0.5 cm) were taken from 30-day-old seedlings, then cultured on solidified MS medium containing different combinations of 2,4-dichlorophenoxy acetic acid (2,4-D) + kinetin (kin). The concentrations of two plant growth regulators were as follows: 2,4-D (1.0, 2.0, and 4.0 mg L^−1^) and kin (1.0 and 2.0 mg L^−1^). After 30 days of culturing, the callus initiation percentage was recorded and calculated based on the following equation:

$$\mathrm{Callus}\;\mathrm{initiation}\;\mathrm{percentage}=(\mathrm{number}\;\mathrm{of}\;\mathrm{initiated}\;\mathrm{calli}/\mathrm{number}\;\mathrm{of}\;\mathrm{inoculated}\;\mathrm{explant})\times100.$$

Initiated calli were subcultured two times to get friable calli, and then, they were used to perform cell suspension cultures.

### Cell suspension culture and growth curve

The friable calli (0.2 g) produced after two sub-cultures on the most convenient medium were transferred into fresh liquid media containing 1.0 mg L^−1^ 2,4-D + 2.0 mg L^−1^ benzyladenine (BA). Cell growth and viable cell number were evaluated using the trypan blue exclusion method [13]. Aliquots containing 150 μL of cell suspension culture were gently mixed with an identical volume of 0.4% (w/v) trypan blue and incubated in the dark for 10 min. A 10-μL sample was observed under a Leica ATC 2000 light microscope, and the number of viable (unstained) and dead (stained) cells was subsequently determined. The viable cell number and viability (%) were calculated using the following equation:

$$\mathrm{Viable}\;\mathrm{cell}\;\mathrm{number}/\mathrm{ml}=(\mathrm{number}\;\mathrm{of}\;\mathrm{counted}\;\mathrm{live}\;\mathrm{cells}/\mathrm{number}\;\mathrm{of}\;\mathrm{squares})\;\times\;\mathrm{dilution}\;\mathrm{factor}\;\times\;10^4;\;\mathrm{viability}\;(\%)=(\mathrm{number}\;\mathrm{of}\;\mathrm{counted}\;\mathrm{live}\;\mathrm{cells}/\mathrm{total}\;\mathrm{counted}\;\mathrm{cells})\;\times\;100$$

### Effect of different amino acid additions on glucosinolates content

The amino acids used in the experiments were filter-sterilized and added to the autoclaved medium for four weeks. In the second passage of cell suspension, 1.0 g L^−1^ of different amino acids (L-phenylalanine, L-tyrosine, L-methionine, and L-cysteine) were added. After that, the callus tissues were isolated from the media, collected, and oven-dried for 24 h at 65 °C. Then glucosinolates and oil fractions were extracted and detected by gas chromatography–mass spectrometry (GC–MS).

### Extraction method and determination analysis

#### Extracts preparation

The extraction method of glucosinolate derivatives was carried out according to Al-Gendy and Lockwood [14]. Oil extracts were carried out as follows: dry 5 g of each suspension cell derived from leaf and root were extracted by adding 250 ml of petroleum ether and heating to 70 °C under reflux for 4 h. The extracts were collected after filtration using Whatman No. 1 filter paper, then evaporated below 40 °C, and stored at 4 °C until further use.

### Gas chromatography–mass spectrometric analysis

The extracts were analyzed in the National Research Center using gas chromatography–mass spectrometry with the following specifications. The GC–MS analysis was performed using a Thermo Scientific, Trace GC Ultra/ISQ Single Quadrupole MS, TG-5MS fused silica capillary column (30 m × 0.251 mm, 0.1-mm film thickness). An electron ionization system with an ionization energy of 70 eV was used for GC–MS detection. Helium gas was used as the carrier gas at a constant flow rate of 1 ml min^−1^. The injector and MS transfer line temperature was set at 280 °C. The sample injection volume was 2 μl. The quantification of identified components was investigated using a relative peak area (%). Tentative identification of the compounds was performed based on the comparison of their relative retention time and mass spectra with those of the NIST, WILLY library data of the GC–MS system.

### Anticancer activity

#### Cell lines

Human breast cancer cells (MCF-7 cell line), human lung cancer cells (A-549 cell line), human colorectal cancer cells (HCT-116 cell line), human liver cancer cells (HepG2), human colon cancer cells (caco2 cell line), human prostate cancer cells (PC3 cell line), human melanoma cancer (A-375 cell line), and a normal human cell line (BJ-1); “immortalized telomerase normal foreskin fibroblast cell line” were obtained from Karolinska Center, Department of Oncology and Pathology, Karolinska Institute and Hospital, Stockholm, Sweden.

#### Cell culture

The procedure was carried out in a sterile area using a laminar airflow cabinet. The culture was maintained in RPMI 1640 medium with 1% antibiotic–antimycotic mixture (10,000 U/mL potassium penicillin, 10,000 ug/mL streptomycin sulfate, and 25 ug/mL amphotericin B),1% L-glutamine, and supplemented with 10% heat-inactivated fetal bovine serum. Doxorubicin was used as a positive control. A negative control, composed of DMSO, was also used. Culturing and sub-culturing were carried out according to Thabrew et al. [15].

#### Cell viability assay

This was done according to Mounier et al. [16]. The cells were seeded at a density of 10 × 10^3^ cells per well in the case of MCF-7 and PC3 and HepG2; 20 × 10^3^ cells per well in case of A-549, HCT-116, caco2, and A-375 cell lines; and 35–45 × 10^3^cells/well in case of BJ-1 using 96-well plates at 37 °C. After 48 h of incubation, the medium was aspirated and 40 uL MTT salt (2.5 mg/mL) was added and further incubated for 4 h. Then, 200 uL of 10% sodium dodecyl sulfate (SDS) was added. The absorbance was measured at 595 nm.

### Determination of IC_50_values

IC_50_ values were calculated using probit analysis and the SPSS computer program (SPSS for windows, statistical analysis software package/version 9/1989 SPSS Inc., Chicago, IL, USA).

### Human CASP3 (Caspase 3) estimation

Quantitative identification of human CASP3 (Caspase 3) using an ELISA Kit from Invitrogen INC. Catalog # KHO1091 (96 tests) (542 Flynn Road, Camarillo, CA 93,012). The procedure was done according to the manufacturer’s instructions.

### Measurement of BCl-2 levels

BCL-2 in the samples and standards were estimated according to Barbareschi et al. [17]. A biotin-conjugated antibody was added, followed by streptavidin-HRP. The reaction was terminated by adding acid, and absorbance was measured at 450 nm.

### Measurement of Bax levels

Bax protein levels were evaluated according to Onur et al. [18]. A monoclonal antibody specific to Bax captured on the plate was added. After incubation, Streptavidin conjugated to Horseradish peroxidase was added. The reaction was then terminated by adding acid and measuring the optical density of the produced color at 450 nm.

### Cell cycle analysis and apoptosis detection

Apoptosis detection and cell cycle analysis were carried out using flow cytometry. HepG2 cells were seeded at 1–5 × 10^4^ and incubated at 37 °C, 5% CO_2_ overnight. After treatment with the tested compound C, for 24 h, cell pellets were collected and centrifuged (300 × g, 5 min). Cell pellets were fixed with 70% ethanol on ice for 15 min for cell cycle analysis and collected again [19]. The collected pellets were incubated with a propidium iodide (PI) staining solution at room temperature for 1 h. Apoptosis detection was performed by Annexin V-FITC apoptosis detection kit (BioVision, Inc, Milpitas, CA, USA) following the manufacturer’s protocol. The samples were analyzed using a FACS Calibur flow cytometer (BD Biosciences, San Jose, CA).

## Results

**Fig. 1:**
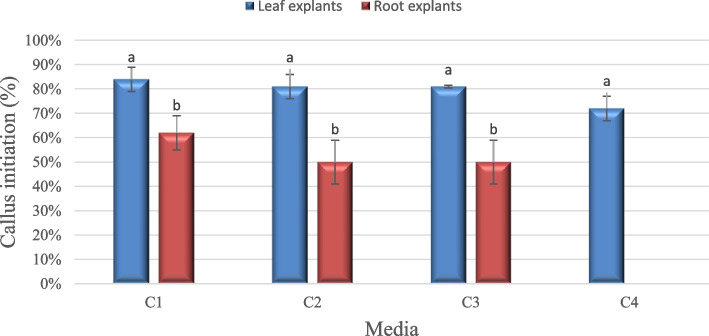
Callus initiation (%) of leaf and root explants cultivated on MS medium and supplemented with different 2,4-D and kin concentrations after 4 weeks of inoculation. While C1(1.0 mg/l 2,4-D + 1.0 mg/l kin), C2(2.0 mg/l 2,4-D + 1.0 mg/l kin), C3(4.0 mg/l 2,4-D + 1.0 mg/l kin), C4(4.0 mg/l 2,4-D + 2.0 mg/l kin). Values = average ± standard error, small letter express LSD significant differences (LSD_0.050_ value = 15.179)

Callus initiation (%) was presented in Fig. 1. Calli was initiated in all 2,4-D and Kin concentrations tested, with the exception of the C4 medium (4.0 mg/l 2,4-D + 2.0 mg/l Kin) used to induce calli from root explant. Leaf explants were more responsive to initiating calli in all 2,4-D + Kin concentrations tested than root explants.

C1 medium (1.0 mg/l 2,4-D + 1.0 mg/l kin) was the most convenient treatment that obtained the maximum callus initiation (%) from two types of explants, recording 84% from the leaf and 62% from the root. Whereas C4 (4.0 mg/l 2,4-D + 2.0 mg/l kin) showed the lowest callus initiation (%), leaf explants recorded 72%, and no calli was initiated from root explants. It was noted that initiating calli from two types of explants required lower concentrations of 2,4-D + kin than higher concentrations. The highest 2,4-D + kin concentrations found in the C4 medium resulted in no calli initiating from root explants accordingly. Moreover, leaf explants respond more strongly to initiating calli than root explants. This could be due to different internal hormone levels in different plant parts.

There is also no significant difference in the leaf or root explant response to varying concentrations of 2,4-D + kin, but there is a significant difference in the level of explant response.

**Fig. 2:**
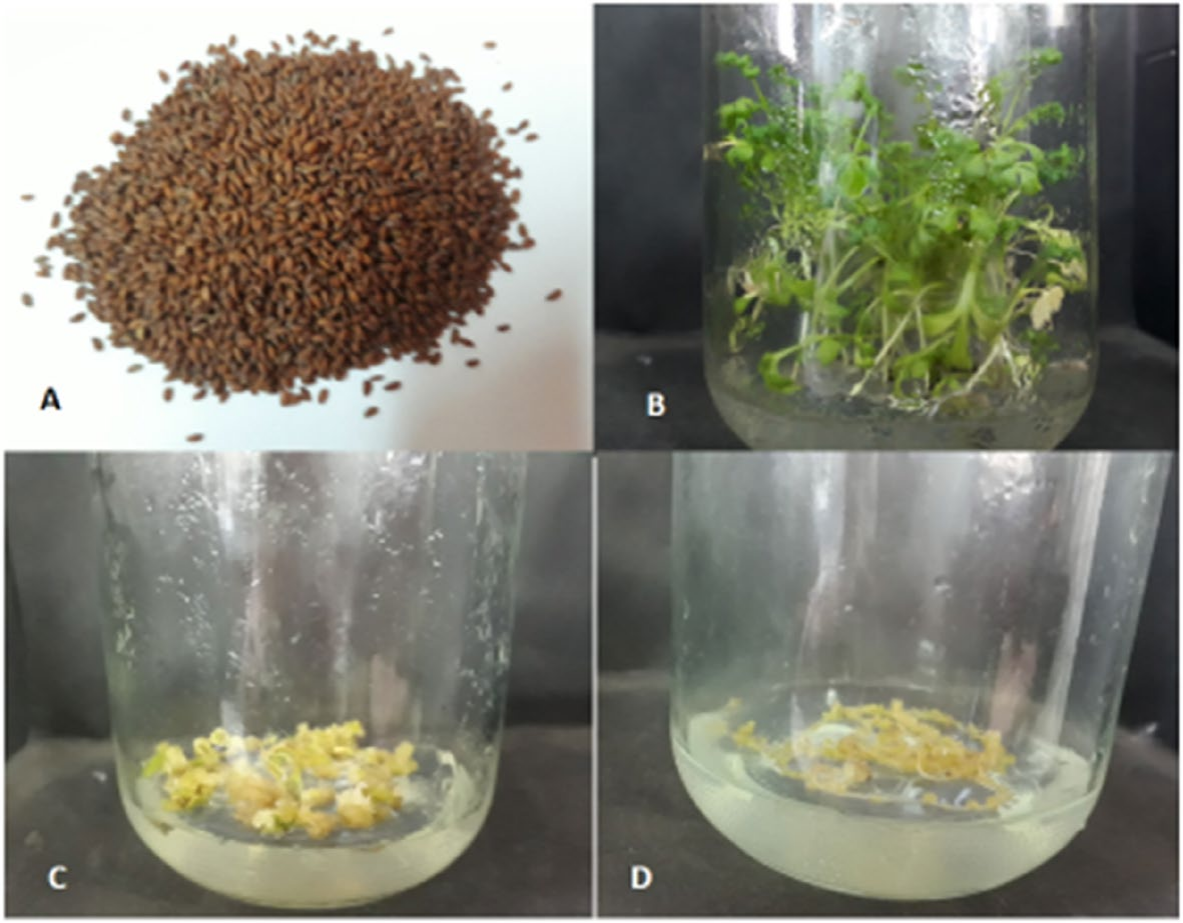
Seeds and seedlings of *L. sativum* (**A** and **B**), callus initiation from leaf explant (**C**), and root explant (**D**) on C1 medium (1.0 mg/l 2,4-D + 1.0 mg/l kin) after 1 month

Other concentrations of growth regulators have been used to induce calli, such as different concentrations of naphthalene acetic acid (0.5, 1.0 mg/l) + benzyl adenine (0.5 mg/l), and they were not effective in callus initiation (data not shown). Callus was grown on a C1 medium and subcultured twice to the same 2,4-D + kin concentration to be used later in cell suspension cultures. Figure 2 shows the seedlings and callus cultures from leaf and root explants on the most convenient medium (C1).

**Fig. 3:**
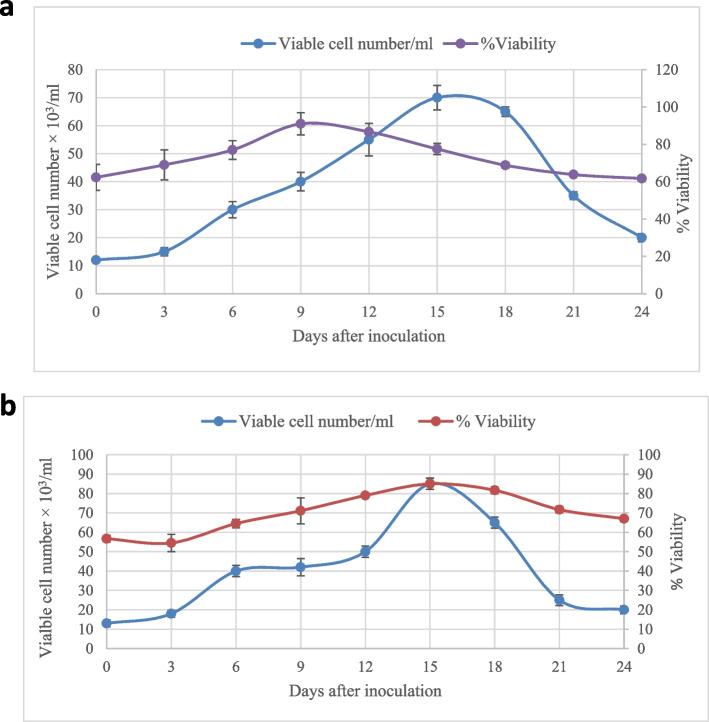
The growth curves, viable cell number, and viability (%) of cell suspension cultures from root (**a**) and leaf (**b**) in the medium containing 1.0 mg/l 2,4-D + 2.0 mg/l BA. Values = average ± standard error

Cell suspension culture was established after two sub-cultures of callus on the solid medium. Both growth curves (Fig. 3a and b) clarify different growth phases; lag phase, log phase, stationary phase, and decline phase. During the log phase (between day 4 and day 15), the viable cell number increased until they reached maximum values on day 15, representing 70 × 10^3^/ml and 85 × 10^3^/ml for both root and leaf cell suspension cultures, respectively. On day 18, the viable cell number decreased (30 × 10^3^, 25 × 10^3^ for root and leaf cell suspension cultures, respectively, in the decline phase). This could indicate a change in cellular metabolism as multiplying cells stop with nutrient decrease. It is clear from the results that the viability of cell suspension cultures produced from leaf explants was higher than that produced from root explants. Also, the fifteenth day of growth recorded the highest vitality in both cell suspension cultures produced from leaf or root. In general, callus growth was appropriate in the suspension cultures. This enabled us to add some precursors that could enhance the active compounds of interest in the study.

**Fig. 4:**
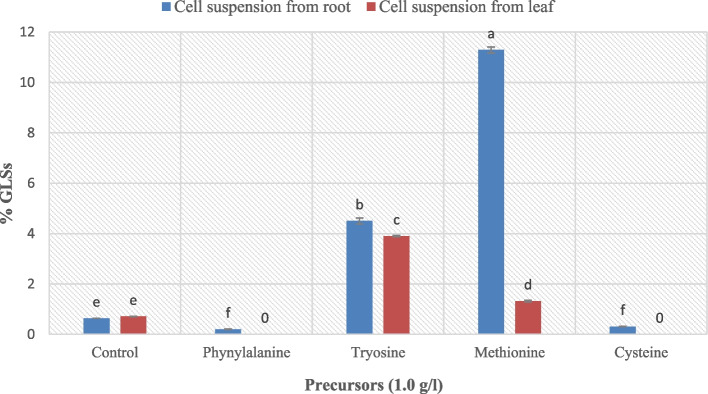
GLSs (%) resulted from different amino acid additions on cell suspension cultures derived from leaf and root. Values = average ± standard error. Small letters express LSD significant differences (LSD_0.050_ value = 0.135)

1.0 g of different commonly used amino acids (precursors) was added to cell suspension cultures derived from both root and leaf. After 1 month, the cells were harvested for glucosinolate (GLSs) extraction and detection. Table 1 shows that glucosinolates or their hydrolysis products were detected in all treatments and the control. The highest percentage was recorded in cell suspensions from roots treated with methionine and tyrosine (11.82% and 4.71%, respectively) (Fig. 4).

**Table 1:** Effect of different amino acid additions on GLSs and GLSs hydrolysis products of cell suspension culture derived from root and leaf after 1 month

Explant-derived calli	Control	L-Penylalanine	L-Tyrosine	L-Methionine	L-Cysteine
Cell suspension derived from root	Thiosulfuric acid S2 [tetra hydro-1,1-dioxido-3-thienyl amino] ethyl] ester	3,6- (1,2-Dicyano etheno)-6-matacyclophane	Benzenesulfonamie N(2,5-dimethylphe-nyl) 4(1-methylethyl)	Hexamethylene diisocyanate	1-Cyano-1,1-dideuterio Hexadecane
	Ethyl- 4-cyano -2-oxo-1-spiro-[4.5]-decane-3-carboxylate		2-(3-Pyridyl)-3-(4-toluenesulfonamid propylazetidine	5-Ethyl-4-n propylthiazole	
Cell suspension derived from leaf	3,4-Dihydroisoquinolin-7-ol, 6-methoxy-3,3- dimethyl-1-methylsulfanyl		1-Pyrrolylmethylene Malonodinitrile	1-Cyano-2-methyl isoindole	
	4,4-Dimethyloxazo [2,3d] triazaine 2,2-dioxide		2-(5-Oxotetrahydro furan-2-yl)-4-phenyl but-2-ene-nitrile	2-(Diethylamino)- 4-dicyano methylene-4,5-dihydrothiazole	
			3,6-(1,2-Dicyano etheno)-6- matacyclophane	(E)3(Tolylmethyl sulfinyl)propene	
			1-H-Cyclopenta-(b) pyridine-3-carbonitrile, 4,5,6,7-tetrahydro- 2-methylthio-4-spiro cyclohexane	1(Benzyloxy)-2-fluoro2phenyl-3-(p-toluene sulfonyloxy) propane

**Fig. 5:**
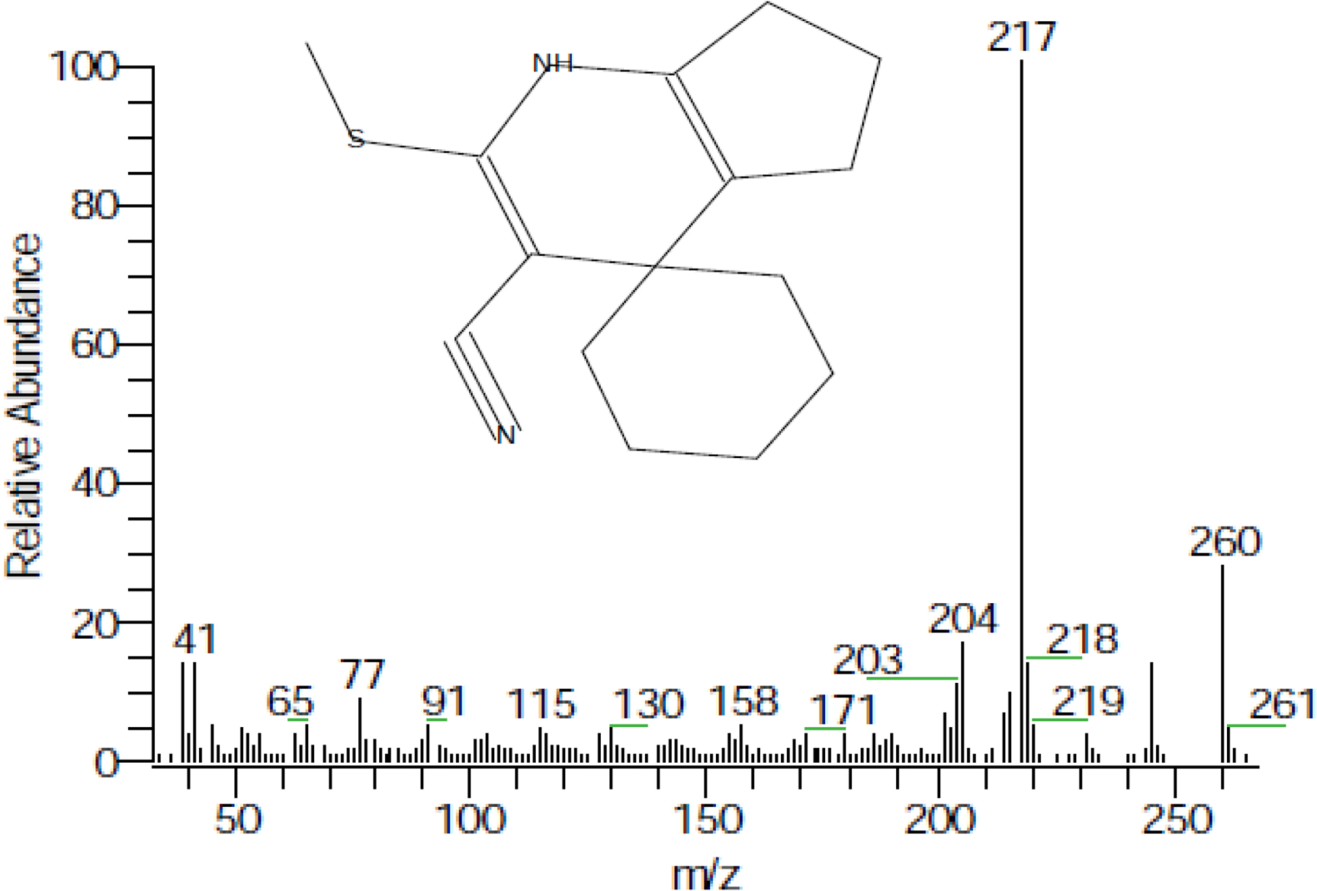
GC–MS chromatogram and structure of 1H-Cyclopenta-(b) pyridine-3-carbonitrile-4,5,6,7-tetrahydro-2-methylthio-4-spirocyclohexane, a major compound detected in cell suspension from leaf treated with L-tyrosine

In terms of the leaf cell suspension, glucosinolates were detected in the cell suspensions treated with L-tyrosine, L-methionine, and control. In contrast, the glucosinolates disappeared with L-phenylalanine and L-cysteine treatments. A maximum percentage of GLSs was recorded with L-tyrosine treatment (3.95%, Fig. 4). The major compound detected in leaf cell suspension treated with L-tyrosine was 1H-Cyclopenta (b) pyridine-3-carbonitrile-4,5,6,7-tetrahydro-2-methylthio-4-spirocyclohexane (3.18%, Table 1 and Fig. 5). In both leaf and root cell suspensions, L-phenylalanine and L-cysteine obtained ineffective results. Generally, it can be mentioned that L-tyrosine and L-methionine amino acids were the most effective on glucosinolate formation in the cell suspension of *Lepidum sativum*. Significant differences exist between treatments and control, as well as within treatments.

Petroleum ether extracts of cell suspension obtained from root and leaf were analyzed using gas chromatography–mass spectrometry and are shown in Table 2. Results clarify that 50 compounds were detected in two oil extracts. The major compounds in root cell suspension were benzene-1,3,5-trimethyl (12.99%), followed by phenyl glyoxal ketoxime (11.66%), formic acid-2-propyl pentyl ester (9.28%), and benzene-1-methyl-2-propyl (5.8%). Other compounds were detected with a lower percentage, for instance, hydrocarbons, different fatty acids, and cyanide compounds (4.23%).

**Table 2:** GC–MS of petroleum ether fraction extracted from cell suspension derived from root and leaf

Compounds	RT	Molecular formula	Peak area (%)	
			Cell suspension from root	Cell suspension from leaf
2-[4-Methyl-6-(2,6,6-trimethyl-cyclo hexenyl)-hexa-1,3,5trienyl]-cyclohex-1-en-1- carboxaldehyde	5.09	C23H32O	1.01	
Serverogenin acetate	5.19	C29H36O10	0.99	
Pregn-5-ene-3,11-dione,17,20,20,21-bis [methylene-bis(oxy)], cyclic-3(1,2-ethanediyl acetal)	5.6	C25H34O7	1.77	
Solasodine	5.9	C27H43NO2	0.58	
Hi oleic safflower oil	6.1	C21H22O11	2.04	
O,O-Diethyl-1-aminopropane phosphonate	6.5	C7H18NO3P	0.93	
Veratroylzygadenine	6.7	C36H51NO10	1.18	
2,2,3,3,4,4-Hexa-deutero-octadecanal	6.9	C18H30D6O	0.89	
Benzene-1,3,5-trimethyl	7.4	C9H12	12.99	4.17
Ethyl-4-hydroxy-2-methylene-4-phenyl-butanoate	7.6	C13H16O3	4.04	
Ethanol- 2,2,2-trichloro	7.8	C2H3Cl3O	2.43	
Phenyl glyoxal ketoxime	8.2	C8H7NO2	11.66	
Octane- 2-methyl	8.6	C9H20	3.26	
Benzene-1-methyl-2-propyl	9.9	C10H14	5.8	
1,3,5-Cycloheptatriene, 7,7-dimethyl	10.0	C9H12	2.80	
4-Ethyl-2-methyl pyrrole-3-carbonitrile	10.3	C8H10N2	1.44	10.45
1H-Indazole	10.9	C7H6N2	4.57	
1-(p-Ethyl phenyl)-2-cyano propane	11.2	C12H15N	2.79	
Trans-p-menthan-1,8-dien-5-acetate	11.5	C12H18O2		1.46
Phenol-2-methyl-6-( 2-propenyl)	11.7	C10H12O	2.31	5.79
Quinoline-5-sulfonic acid, 8-methoxy, (2,4,6-trimethylphenyl) amide	11.9	C19H20N2O3S		4.07
4-Methyl amino benzoic acid, 4-formyl phenyl ester	12.1	C15H13NO3	2.43	
Benzene-2-ethyl-1,4-dimethyl	12.4	C10H14	2.36	10.66
Benzene-ethanamine, 2,5-dimethoxy-à-4-dimethyl	12.6	C12H19NO2		0.97
9-Methyl-6-(1-naphthyl methyloxy)-4-ethyl-á-carboline-3-carboxylic acid ethyl ester	13.5	C28H26N2O3		9.66
Naphthalene	13.8	C10H8		8.51
2-H-1-Benzopyran-2-one,3,4 Dihydro	14.0	C9H8O2		1.54
Decane- 2-methyl	14.5	C11H24		5.52
1,3-Diethyl-2-methyl-1,3 diazolidine	17.0	C8H18N2	3.52	
Naphthalene- 2-methyl	17.5	C11H10		9.23
3-Chloro-methyl-heptane	19.5	C8H17Cl		1.85
Naphthalene- 1-ethyl	19.8	C12H12	1.04	
9,9-a-Dihydro-3H-pyrrolo[1,2-a] indole	20.5	C11H11N		2.50
Formic acid-2-propyl pentyl Ester	24.3	C9H18O2	9.28	
4,4-Dimethyl1octene	24.8	C10H20	0.97	1.83
Heptadecane	26.5	C17H36	1.38	
Octadecane	28.5	C18H38	1.25	1.44
3-Methyl-2-butenyl-2-methyl-3-oxobutanoate	30.5	C10H16O3	1.16	
Nonadecane	30.9	C19H40		1.62
Pentadecanoic acid-14-methyl, methyl ester	31.0	C17H34O2	3.23	
Hexadecanoic acid methyl ester	31.3	C17H34O2		2.13
Undecane	32.2	C11H24	0.94	1.15
Eicosane	32.4	C20H42		0.94
Tetradecane	34.2	C14H30	0.75	1.90
Triacontane	34.4	C30H62		1.18
9,12-Octadecadienoic acid-(Z,Z), methyl ester	34.8	C19H34O2	1.36	
9-Octadecenoic acid (Z), methyl ester	35.0	C19H36O2	2.54	0.87
Heptane- 2,4,6-trimethyl	35.9	C10H22	0.57	
Nonacosane	37.5	C29H60	0.40	0.62
Tricosane	37.9	C23H48		0.40

**Fig. 6:**
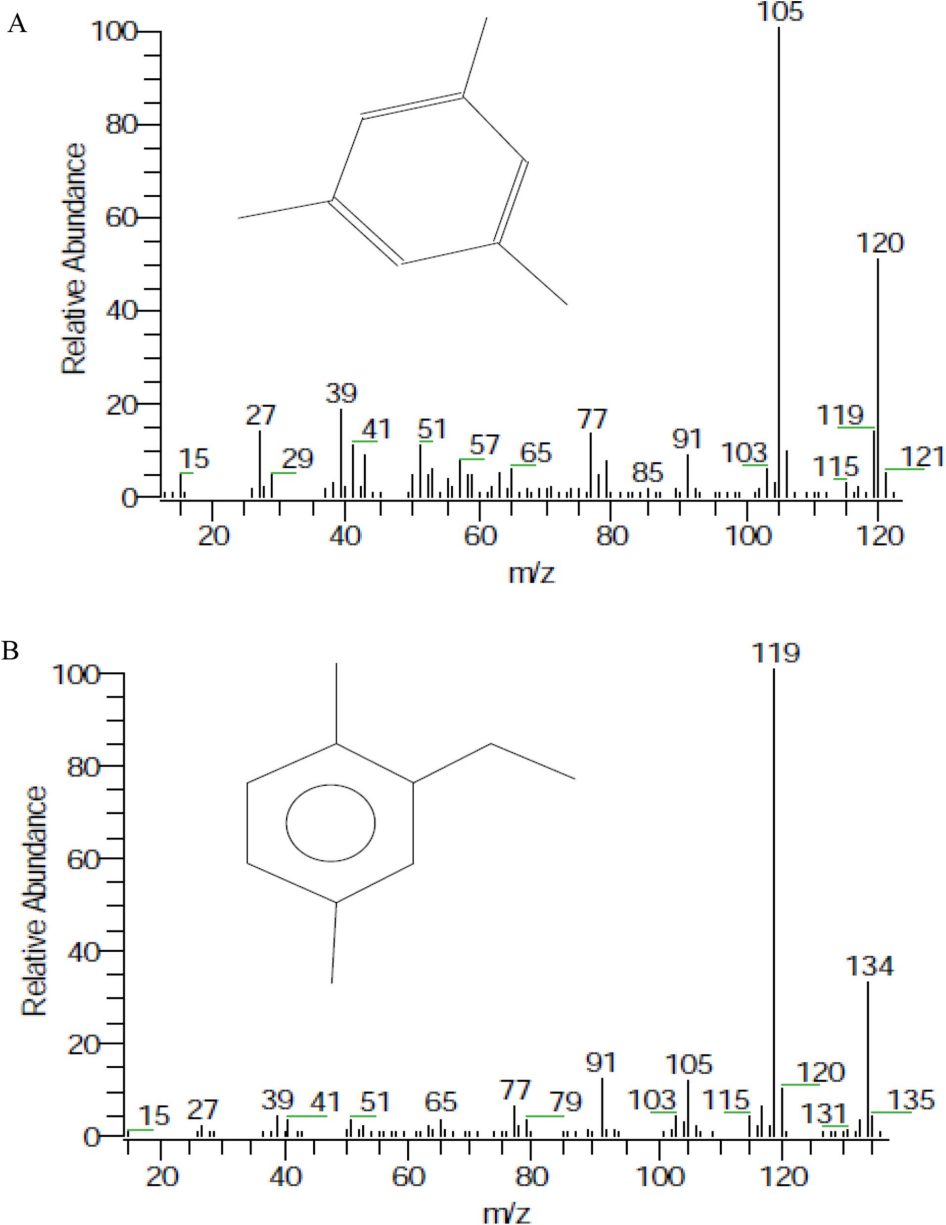
GC–MS chromatograms and structures of benzene-1,3,5-trimethyl (**A**), and benzene-2-ethyl-1,4-dimethyl (**B**), the major compounds in cell suspension derived from root and leaf, respectively

While in the extract of leaf cell suspension, the major compounds were benzene-2-ethyl-1,4-dimethyl (10.66%), followed by 4-ethyl-2-methyl pyrrole-3-carbonitrile (10.45%), 9-methyl-6-(1-naphthyl methyloxy)-4-ethyl-á-carboline-3-carboxylic acid ethyl ester (9.66%), naphthalene-2-methyl (9.23%), and naphthalene (8.51%). Figure 6A and B show the chromatograms and structures of the major compounds detected in both cell suspensions. The same compounds were detected in leaf cell suspension, which are expected, hydrocarbons, different fatty acids, and cyanide compounds (10.45%). Additionally, it was found that some steroid compounds and derivatives of formic acid compounds are present in root cell suspensions but not in leaf extract. Quinoline-5-sulfonic acid, 8-methoxy, (2,4,6-trimethylphenyl) amide (4.07%) was only detected in leaf cell suspension (a derivative of lepedine, a major compound in *Lepidium sativum*, and it has a variety of biological effects). This highlights the necessity of creating plant cell cultures from different explants and shows that the range of chemicals that can be recovered in the produced extracts may vary depending on the choice of grown explant.

### Anticancer activity

Eight samples were tested for their anti-proliferative activity against seven human cancer cell lines at 100 ug/ml, as shown in Table 3. Glucosinolate extracts from root cell suspension treated with L-tyrosine (GRT) showed the highest anti-proliferative responses on PC3, A-549, caco2, and HepG2 cell lines compared to the other glucosinolate extracts from root cell suspension and root cell suspension treated with L-methionine (GR and GRM). While all three glucosinolate extracts from root cell suspensions (GR, GRM, and GRT) gave weak cytotoxic responses on A-375, MCF-7, and HCT-116 cell lines. Unlike the three glucosinolate extracts from leaf cell suspensions (GL, GLM, and GLT), which showed significant anticancer effects on PC3, caco2, and HepG2 cell lines, only glucosinolates from leaf cell suspension (GL) gave a promising impact on the A-549 cell line, while they showed weak activity on MCF-7. The activity was slightly increased when cell suspension was treated with L-methionine and L-tyrosine, which recorded 44.8 and 58.6%, respectively. According to our results, it was observed that treating root cell suspension with L-tyrosine significantly increased the anticancer activity of glucosinolate extracts on the prostate (PC3), lung (A-549), colorectal (caco2), and hepatocellular (HepG2) carcinoma cell lines. The petroleum ether fractions from root and leaf cell suspension (OR and OL) demonstrated a profound anticancer effect on five cell lines (MCF-7, PC3, A-549, caco2, and HepG2), which was greater than its cytotoxic effect in the remaining two cell lines (A-375 and HCT-116). These remarkable responses of glucosinolates and their treatment encourage us to test their specificity and selectivity. Further screening appeared on the normal human skin fibroblast cell line (BJ-1) at the same concentrations as on overall human tumor cancer cells, as shown in Table 3.

**Table 3:** In vitro screening of the antiproliferative activities of tested extracts against different carcinoma cell lines

Extracts	Cytotoxicity % at 100 ug/ml							
	A-375	MCF-7	HCT-116	PC3	A-549	caco2	HepG 2	BJ-1
GR	0.0 ± 0.03	0.0 ± 0.1	8.2 ± 0.7	2.0 ± 0.3	19.2 ± 0.7	25.5 ± 0.5	38.9 ± 0.8	0.0 ± 0.2
GRM	0.0 ± 0.01	6.0 ± 0.3	0.0 ± 0.4	7.8 ± 0.6	17.5 ± 0.4	20.1 ± 0.7	46.3 ± 1.3	0.0 ± 0.04
GRT	0.0 ± 0.02	0.0 ± 0.2	0.0 ± 0.2	73.9 ± 1.3	58.9 ± 1.4	58.0 ± 1.4	89.4 ± 1.1	0.0 ± 0.1
GL	18.9 ± 0.7	18.3 ± 0.4	18.5 ± 0.4	83.4 ± 0.9	71.7 ± 1.5	74.2 ± 0.9	94.4 ± 1.4	9.5 ± 0.4
GLM	25.8 ± 0.5	44.8 ± 0.7	33.5 ± 1.2	92.6 ± 2.2	59.4 ± 2.2	87.9 ± 2.1	98.2 ± 2.1	23.3 ± 0.3
GLT	0.0 ± 0.02	58.6 ± 1.1	0.0 ± 0.3	86.8 ± 0.9	35.2 ± 0.7	80.9 ± 2.1	99.8 ± 0.7	0..0 ± 0.1
OR	8.8 ± 0.6	96.6 ± 2.1	49.3 ± 0.6	97.2 ± 1.1	97.2 ± 1.4	79.3 ± 0.7	71.1 ± 2.3	68.2 ± 1.4
OL	0.0 ± 0.4	77.5 ± 1.7	46.7 ± 1.4	91.2 ± 1.7	94.6 ± 2.4	92.0 ± 1.5	60.3 ± 2.4	98.0 ± 2.3
Doxorubicin	99.9 ± 2.1	88.7 ± 1.9	72.0 ± 2.4	84.4 ± 1.8	70.0 ± 1.7	99.0 ± 2.6	90.0 ± 2.7	89.1 ± 2.2

Values = average ± standard deviation for the tested extracts; GR glucosinolates of root cell suspension, GRM glucosinolates of root cell suspension treated with L-methionine, GRT glucosinolates of root cell suspension treated with L-tyrosine, GL glucosinolates of leaf cell suspension, GLM glucosinolates of leaf cell suspension treated with L-methionine, GLT glucosinolates of leaf cell suspension treated with L-tyrosine, OR petroleum ether extract of root cell suspension, OL petroleum ether extract of leaf cell suspension

According to our findings, all glucosinolates from the leaf and root cell suspension were safe on the tested normal human cells, in contrast to the petroleum ether fractions, which were toxic on the tested normal cells.

To distinguish between all glucosinolate extracts that showed 60% cytotoxicity on the tested cell lines and determine their order in cytotoxicity, IC_50_ was calculated using a second assay in which each active extract was screened at four additional concentrations (100, 50, 25, and 12.5 ug/ml) as shown in Table 4. According to our results, GL extract was selected among all glucosinolates to further study their mechanistic effects on hepatocellular cancer cells (HepG2) due to their safety and potency, with a potent IC_50_ value on HepG2 with no toxicity on normal cell tested. The apoptotic mode of action of GL on HepG2 was studied. Also, different apoptotic parameters were studied, including BAX, BCL2, Caspase3, and cell cycle analysis.

**Table 4:** IC_50_ of selected extracts exhibiting more than 60% cytotoxicity

Extracts	IC_50_ (ug/ml)				
	MCF-7	pc3	A-549	caco2	HepG2
GRT	––	72.4 ± 0.8	92.6 ± 1.3	89.9 ± 2.6	61 ± 1.1
GL	––	59.7 ± 1.3	77.1 ± 0.7	56.6 ± 2.2	47.5 ± 1.9
GLM	––	51.4 ± 1.6	––	59.5 ± 2.3	38.5 ± 0.3
GLT	––	59.4 ± 2.1	––	68.7 ± 2.2	41.9 ± 0.8
OR	61 ± 0.9	66.2 ± 1.7	42.3 ± 1.1	72 ± 2.5	81.2 ± 0.7
OL	71.1 ± 1.4	54.4 ± 1.3	48 ± 0.7	63.6 ± 2.4	––

Values = average ± standard deviation for tested extracts; GRT glucosinolates of root cell suspension treated with L-tyrosine, GL glucosinolates of leaf cell suspension, GLM glucosinolates of leaf cell suspension treated with L-methionine, GLT glucosinolates of leaf cell suspension treated with L-tyrosine, OR petroleum ether extract of root cell suspension, OL petroleum ether extract of leaf cell suspension

### Cellular mechanism of action

**Fig. 7:**
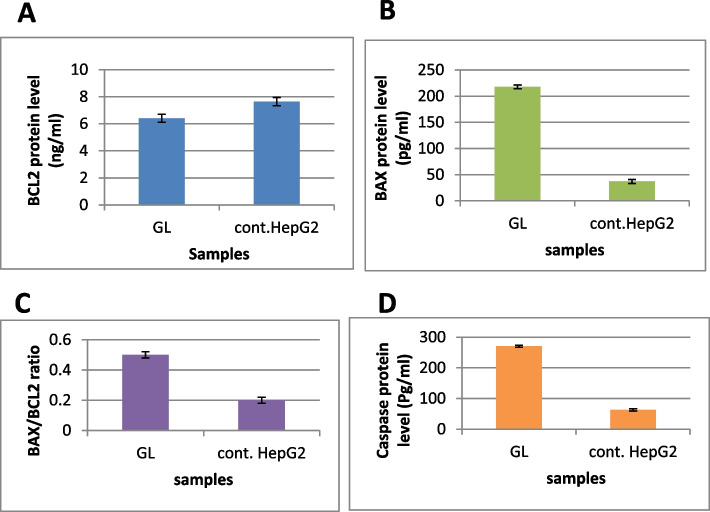
Quantitative analysis of Bax, BCL-2, Bax/BCL-2 ratio, and caspase-3 for HepG2 treated with IC_50_ (47.5 ug/ml) of GL extract compared to untreated HepG2 (*p* < 0.05), where the *Y*-axis represents the protein levels. Values = average ± standard deviation. GL, glucosinolates of leaf cell suspension

HepG2 carcinoma cell line was treated with 47.5 ug/ml GL extract (the IC_50_ value) to determine the level of BCL2 and BAX compared to the non-treated HepG2 cells, as shown in Fig. 7A and B. From the results, it is clarified that GL extract down-regulates the anti-apoptotic protein BCL2 and up-regulate the pro-apoptotic protein BAX, in addition to disrupting the BAX/BCL2 ratio, which triggers an apoptotic cascade inside liver cancer cells, as shown in Fig. 7C. Moreover, this extract increases caspase 3 level in HepG2 cells from 62.99 to 270.4 Pg/ml as compared to untreated cells, as shown in Fig. 7D.

### Cell cycle arrest

According to Fig. 7, GL extract significantly increased late/secondary cellular apoptosis from 0.19 to 6.34% compared to the untreated HepG2 cells. Also, the GL extract increased the early/primary apoptosis from 0.72 to 1.91%. These results confirmed the apoptotic effect of GL extract on HepG2 cells.

**Fig. 8:**
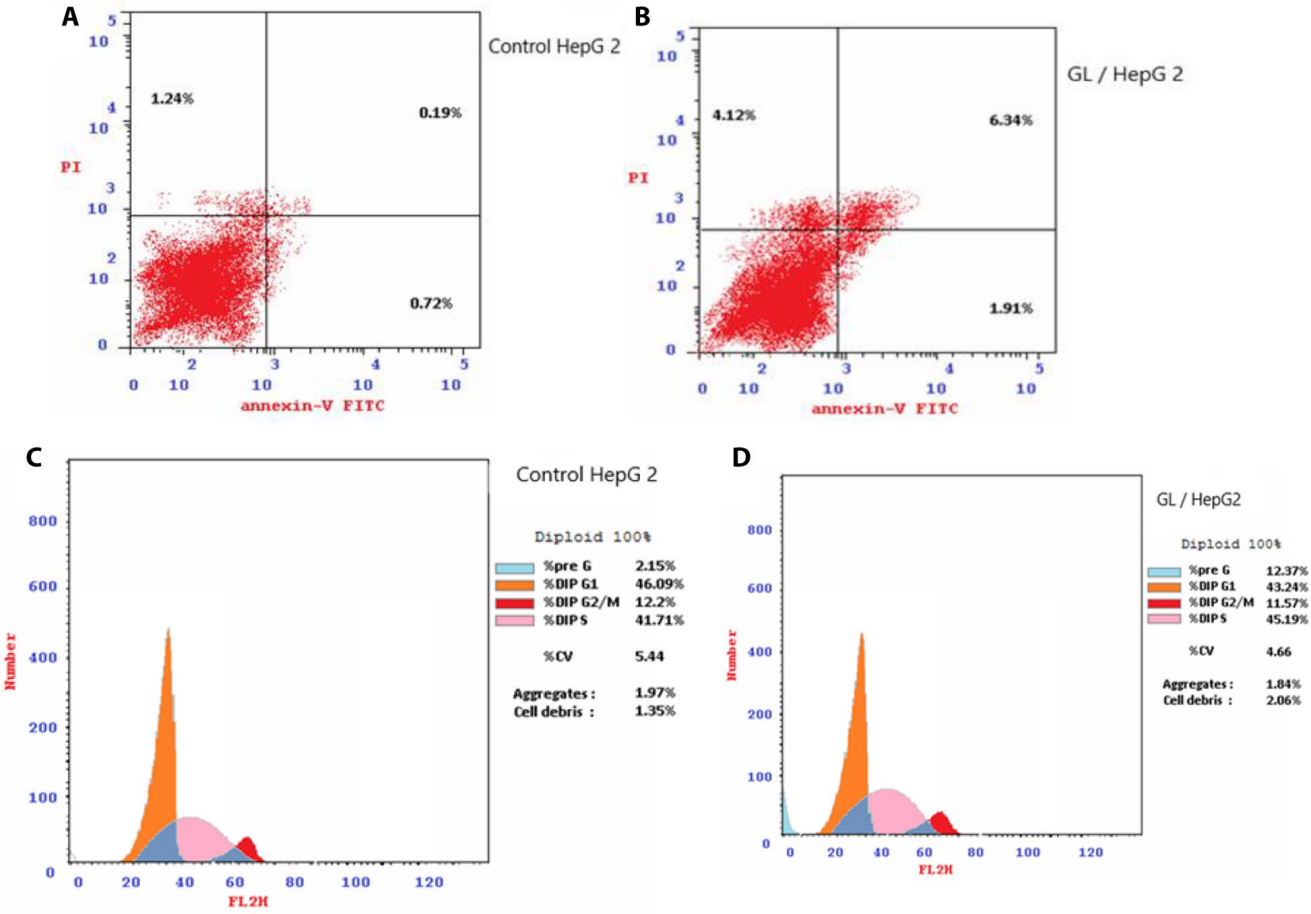
Cellular mechanism of action of GL extract, induction of apoptosis by GL extract (**A**, **B**), the percentage of cells undergoing apoptosis is defined as the sum of early apoptotic (annexin V + /PI −), cell percentage and late apoptotic (annexin V + /PI +), cell cycle analysis of HepG2 after incubation with compound GL for 24 h, untreated cells were used as a control (**C**, **D**)

Further investigation was carried out on GL extract to explore the molecular mechanism through which it exerts its anti-proliferative activity against HepG2 cells. Flow cytometry was used to identify its effect on cell cycle distribution. After treating the HepG2 cells with GL extract at a concentration of 47.5 ug/ml. GL extract signals a significant elevation in the percentage of cells at the pre-G1 phase by 5.7 folds compared with the untreated cells (control) (Fig. 8). Also, GL extract caused the accumulation of cells in the S-phase by 1.1 folds. These results revealed that glucosinolates from leaf suspension cultures (GL extract) inhibited cell proliferation through the induction of S-phase arrest, which led to cell cycle cessation at the S phase.

## Discussion

The current study’s findings were supported by a prior study that found leaf explants more capable of developing calli than hypocotyl and root explants [20]. Additionally, supplemented MS-medium with 2,4-D (1 mg L^-1^) and BAP (2 mg L^-1^) was used to boost the formation of callus and secondary metabolites from *L. sativum* [21].

Several studies found that the availability of free amino acids, such as L-phenylalanine, was increased by introducing L-phenylalanine biosynthetic genes from *Escherichia coli* into *A. thaliana* resulted in increased levels of benzyl GLSs. Additionally, more aliphatic GLSs result from more L-methionine [22, 23].

As an alternative to growing plants in vivo, plant cell suspension culture offers a viable method for producing high-value plant metabolites. Safety and regulatory criteria are introduced with this production technique. Additionally, it is likely to create novel compounds that are not found in native plants [24, 25]. Additionally, plant cell physiology and biochemistry research use cell suspension culture.

Numerous experiments have attempted to produce GLSs in cell cultures. Indian cress (*Tropaeolum majus*) cell cultures produced the most GLSs after substrate feeding, which resulted in 44 mmol/g DW [26]. In hairy root cultures of *Tropaeolum majus*, L-phenylalanine and L-cysteine feeding increased the concentration of GLSs (85.8 mmol/g FW) [27]; however in the current investigation, those two amino acids had less of an impact. The promoting effect of precursor addition on glucosinolate accumulation was also reported in cell suspension cultures of *Nasturtium montanum* and *Cleome chelidonii* [28].

*Lepidium sativum* plants contained thiocyanate-forming protein (TFP) and glucosinolate hydrolysis products [29]. No prior studies have fed amino acids to *L. sativum* cell culture in an attempt to produce glucosinolates. The current study’s findings support the viability of this production, although more research is required to produce more accurate findings. *Lepidium sativum* also contains a variety of active substances in its seed, leaves, and aerial portions [30].

The previous studies isolated and fractionated glucosinolates from *L. sativum* seeds and fresh herbs. They detected 2-ethyl butyl glucosinolate, methyl glucosinolate, and glucotropaeolin in the fresh herbs, in addition to dimeric imidazole alkaloids such as lepidine that were also detectable [5].

Cancer is characterized by cellular changes that result in uncontrolled growth. Even though several treatments and interventions, including chemotherapy, radiation, gene therapy, hormone therapy, immunotherapy, and targeted therapy, have been successfully used to combat cancer, it is still a significant health issue. Even with the most cutting-edge anticancer therapies, several problems remain, such as side effects, cancer resistance, metastasis, and recurrences. Therefore, it is essential to look for new chemotherapeutic agents [31]. Previous studies on the impact of glucosinolates on cancer cells include several mechanisms. The most important is maintaining low levels of systemic oxidative stress, inhibiting angiogenesis and cell cycle progression, and promoting apoptosis of cancerous cells [32]. In addition, it inhibits NF-B signaling, which activates inflammation-related genes such as chemokines, cytokines such as IL-1, IL-6, IL-12, and TNF-, and adhesion molecules [33].

Apoptosis is one of the most effective ways to eliminate cancer cells. The search for novel natural compounds that can induce apoptosis is attracting much interest in cancer research. In our study, glucosinolates from leaf and root cell suspension cultures of *Lepidium sativum* were investigated for their cytotoxic potential against seven human cancer cell lines, including; MCF-7 cell line, A-549 cell line, HCT-116 cell line, HepG2 cell line, caco2 cell line, PC3 cell line, and A-375 cell line. Glucosinolates were further screened at different concentrations to calculate their IC_50_. All extracts were assayed over normal human cells (BJ-1 cell line) to study the safety of glucosinolates. In this study, we examined the anticancer effect of all samples using the MTT assay, which is a reliable assay for studying cellular metabolic activity and mitochondrial dysfunction. As a result, when comparing glucosinolates from different extracts, cell suspension derived from leaf (GL extract) was most effective on HepG2 cancer cells in a dose-dependent manner, so it was subjected to study their possible apoptotic mechanism through their effect on BCL2, BAX, and caspase 3.

Our results revealed that GL extract downregulates the anti-apoptotic protein BCL2 and upregulates the pro-apoptotic protein BAX, a key pro-apoptotic molecule in the mitochondria-dependent apoptotic pathway, and disrupt the BAX/BCL2 ratio, which signals an apoptotic cascade inside HepG2 cancer cells. HepG2 cancer cells treated with GL extract upregulate capasase 3 protein compared to the untreated HepG2 cancer cells.

As cancer is thought to be a cell cycle disease, cell cycle blockage is considered an attractive strategy to fight cancer [34], so we studied the effect of GL extract on the cell cycle progression and induction of apoptosis in HepG2 cancer cells. Flow cytometry was carried out using annexin-V-FITC and propidium iodide (PI) in HepG2 cells. After treatment with IC_50_ concentrations of GL (47.5 ug/ml) for 24 h, the cells were labeled with the two dyes. The corresponding red (PI) and green (FITC) fluorescence were detected with the flow cytometry. The findings revealed that GL extract cytotoxic activity against HepG2 cancer cells was related to S-phase cycle arrest.

## Conclusions

Utilizing components from cell suspension culture combined with precursor feeding has proven to be a successful method for obtaining special compounds with critical industrial uses. L-Tyrosine and L-methionine additions to the cell suspension culture improved the production of glucosinolates in *Lepidium sativum*. Since cancer cannot be cured, additional study is needed to find compounds that can fight it without harming human health, in contrast to the petroleum ether fractions from two suspension cultures, which had a lethal effect on the normal cells. In our study, all glucosinolates extracted from cell suspensions influenced cancer cells while not impacting healthy cells. Understanding cancer biology has become essential for obtaining compounds that fight cancer cells, and it has become the focus of recent studies. Apoptosis is one of the crucial control mechanisms used to combat cancer cells. The glucosinolates extract from our study’s *L. sativum* cell suspension demonstrated its anticancer efficacy, promoted apoptosis, and reduced hepatocellular growth by inducing S phase arrest.

## Data Availability

The data that support the findings of this study are available on request from the corresponding author. The data are not publicly available due to privacy or ethical restrictions.

## Abbreviations

2,4-D: 2,4-Dichlorophenoxy acetic acid
kin: Kinetin
GLSs: Glucosinolate compounds
MCF-7: Human breast carcinoma cell line
A-549: Human lung carcinoma cell line
HCT-116: Human colorectal carcinoma cell line
HepG2: Human liver carcinoma cell line
caco2: Human colon cancer cell line
PC3: Human prostate cancer cell line
A-375: Human melanoma cell line
BJ-1: Normal human cell line
GR: Glucosinolates of root cell suspension
GRM: Glucosinolates of root cell suspension treated with L-methionine
GRT: Glucosinolates of root cell suspension treated with L-tyrosine
GL: Glucosinolates of leaf cell suspension
GLM: Glucosinolates of leaf cell suspension treated with L-methionine
GLT: Glucosinolates of leaf cell suspension treated with L-tyrosine
OR: Petroleum ether extract of root cell suspension
OL: Petroleum ether extract of leaf cell suspension
